# Temporal relationship between malnutrition and oral function impairment in older adults with dysphagia: A cross-lagged panel model

**DOI:** 10.1016/j.jnha.2025.100577

**Published:** 2025-05-06

**Authors:** Hiroyasu Furuya, Takeshi Kikutani, Yuri Yokota, Maiko Ozeki, Fumiyo Tamura

**Affiliations:** a The Nippon Dental University, Tama Oral Rehabilitation Clinic, 4-44-19 Higashi-cho, Koganei-shi, Tokyo 184-0011, Japan; b Division of Rehabilitation for Speech and Swallowing Disorder, The Nippon Dental University Hospital, 2-3-16 Fujimi, Chiyoda-ku, Tokyo 102-8158, Japan

**Keywords:** Malnutrition, Tongue pressure, Dysphagia, Cross-lagged panel model, Older adults

## Abstract

**Objectives:**

This study aimed to longitudinally investigate the temporal relationship between tongue pressure and malnutrition in older adults with dysphagia and to determine the antecedent factors.

**Design:**

This is a retrospective cohort study.

**Setting and Participants:**

In total, 177 participants aged ≥65 years with dysphagia who visited a specialized dysphagia rehabilitation clinic between 2014 and 2018 were enrolled.

**Measurements:**

Malnutrition was assessed based on the phenotypic criteria (unintentional weight loss, low body mass index, and reduced skeletal muscle mass) from the Global Leadership Initiative on Malnutrition framework. Tongue pressure was measured using a tongue pressure measuring device. The bidirectional association between tongue pressure and malnutrition was examined, adjusting for age, sex, cognitive function, occlusal support status, and comorbidities.

**Results:**

In the Cross-Lagged Panel Model, a significant cross-lagged effect was observed from tongue pressure to malnutrition at 6 months (β = −0.135, p < 0.001) and 12 months (β = −0.112, p = 0.028). However, the pathway from malnutrition to tongue pressure was not significant. Logistic regression analysis also revealed that baseline tongue pressure was significantly associated with malnutrition at 6 months (odds ratio [OR] = 0.91, 95% confidence interval [CI]: 0.86–0.95) and 12 months (OR = 0.89, 95% CI: 0.84–0.94). During the follow-up period, tongue pressure improved; however, the prevalence of malnutrition increased.

**Conclusions:**

Decreased tongue pressure may precede malnutrition in older adults with dysphagia; however, a reverse relationship was not observed. The findings suggest the importance of incorporating oral function assessment as part of the risk assessment for malnutrition.

## Highlights

• Low tongue pressure was significantly associated with malnutrition risk at 6 and 12 months.
• No significant path was found from malnutrition to tongue pressure decline.
• Integrate oral function assessment in nutrition care for older adults with dysphagia.

## Graphical abstract

**Figure:**
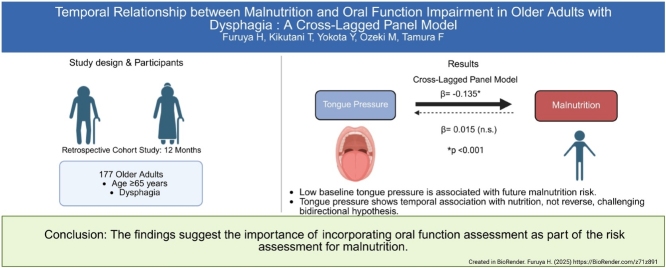

## 1 Introduction　　

Malnutrition in older adults leads to a decline in physical function and sarcopenia owing to weight loss and inadequate energy intake [1]. These consequences collectively contribute to a reduced quality of life and increased mortality risk [2,3]. Malnutrition prevalence ranges from 5−20% in community-dwelling older adults [4,5] to 15–30% in nursing homes and hospitals [6,7], increasing with care dependency. Among various contributing factors [8,9], dysphagia and impaired oral function are major contributors to reduced food intake, exacerbating malnutrition [10].

The relationship between oral function and malnutrition has garnered increasing attention recently. Studies have demonstrated that reduced tongue pressure directly affects chewing ability and swallowing, resulting in decreased food intake and an increased risk of malnutrition [11,12]. Conversely, malnutrition may secondarily weaken the perioral and tongue muscles through systemic sarcopenia [13,14]. Additionally, recent studies have reported associations between frailty and oral function [15] and between sarcopenia and oral function [16]. These associations fit within broader frailty models, where oral dysfunction may both contribute to and result from the cycle of declining physical function and nutritional status. Thus, the relationship between tongue pressure and malnutrition is presumed to be bidirectional, although it remains unclear which factor precedes the other.

Most prior studies have been cross-sectional [17,18]. Only a limited number of studies have investigated the temporal relationship between tongue pressure and malnutrition [19]. Specifically, there is a lack of evidence to guide clinical decisions regarding whether to prioritize oral function interventions or nutritional management, particularly in older adults with dysphagia. Older adults with dysphagia have significantly higher rates of malnutrition and reduced tongue pressure compared to healthy older adults, which can be observed over relatively short periods [20,21]. Therefore, developing effective intervention strategies for this population represents a critical clinical challenge, and examining the temporal relationship between these factors is particularly valuable.

This study aimed to longitudinally examine the bidirectional relationship between tongue pressure and malnutrition in older adults with dysphagia. Using the Cross-Lagged Panel Model (CLPM), both (1) the pathway through which decreased tongue pressure leads to malnutrition and (2) the pathway through which malnutrition leads to reduced tongue pressure were simultaneously assessed. It was anticipated that these findings would offer valuable insights into clinical decision-making, particularly in determining whether to prioritize oral function training, nutritional management, or both in older adults with dysphagia. Furthermore, these findings may aid in developing effective strategies for preventing malnutrition and managing dysphagia in this population.

## 2 Materials and methods

### 2.1 Study design and ethical approval

This retrospective cohort study analyzed electronic health records of adults aged ≥65 years who first visited a dysphagia-specialized oral rehabilitation clinic in Tokyo between April 2014 and March 2018. This study followed the STROBE guidelines for observational studies. The study protocol was approved by the ethics committee of The Nippon Dental University (Approval No.: NDU-T2017-41) and conducted based on the ethical principles outlined in the Declaration of Helsinki

### 2.2 Participants

**Fig. 1:**
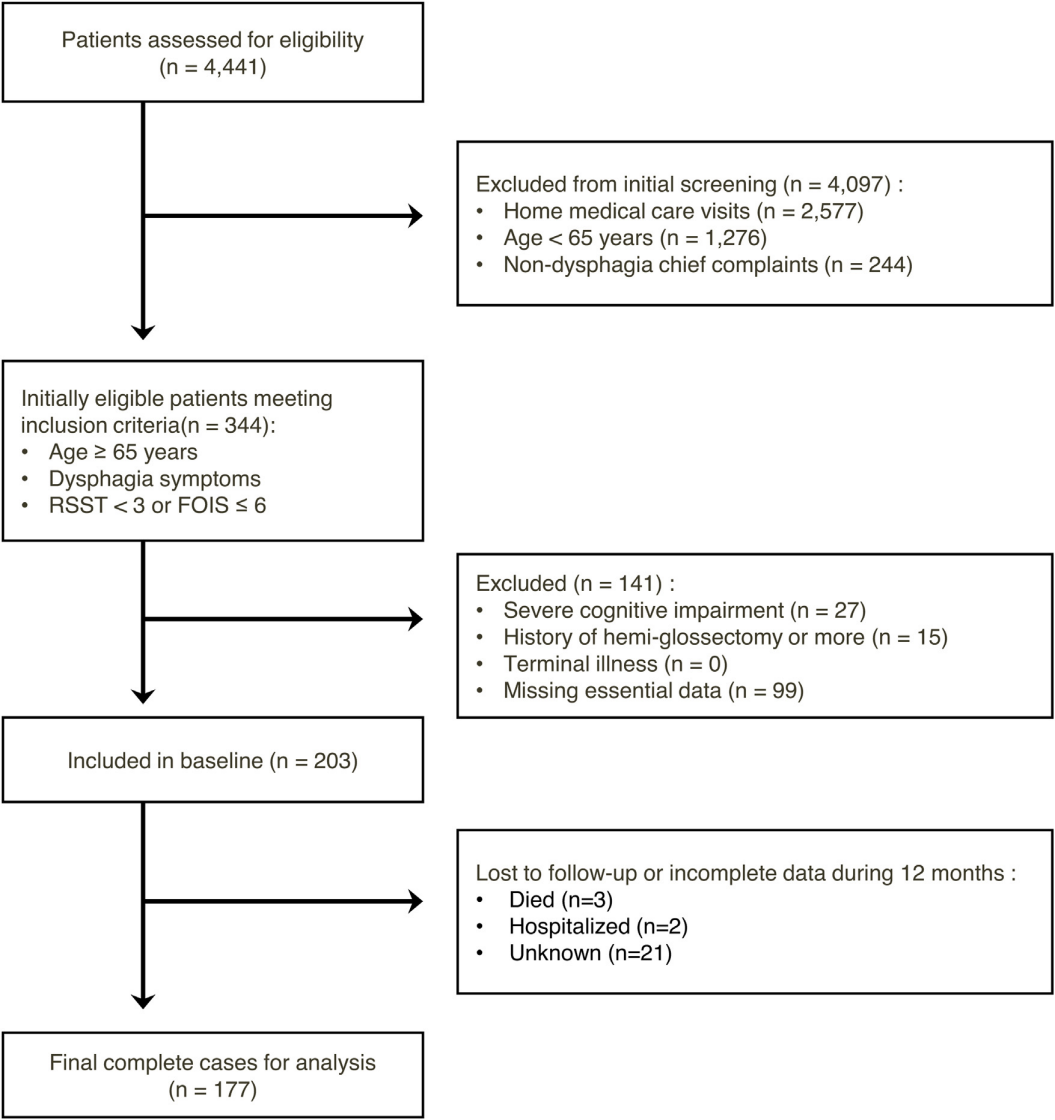
Flowchart of participant selection.

The participant selection process is illustrated in Fig. 1.

The inclusion criteria were: ≥65 years, a complaint of dysphagia, and either a Repetitive Saliva Swallowing Test (RSST) score [22] of <3 or a Functional Oral Intake Scale (FOIS) level [23] of ≤6.

Dysphagia, the impairment of swallowing safety or efficiency, was defined using RSST <3 or FOIS ≤6. Notably, FOIS level 6 indicates patients who avoid specific foods despite not requiring texture modification, which is considered a clinically significant limitation.　

RSST is a non-invasive screening tool that measures the number of saliva swallows in 30 seconds, with <3 indicating abnormal swallowing function. The exclusion criteria were severe cognitive impairment (Mini-Mental State Examination score ≤10), a history of resection involving at least half of the tongue, a terminal illness with a life expectancy of ≤6 months, or missing data for an essential baseline assessment. In total, 177 participants with complete data on tongue pressure and malnutrition at all three time points (baseline, 6 months, and 12 months) were included in the final analysis. All participants provided opt-out consent in accordance with institutional ethical guidelines.

### 2.3 Measurements

The primary outcome measures were malnutrition and tongue pressure.

#### 2.3.1 Malnutrition

Malnutrition was defined based on the phenotypic criteria of the Global Leadership Initiative on Malnutrition (GLIM) [24]. Specifically, participants were classified as malnourished if they met any of the following:

1 Unintentional Weight Loss: ≥5% within 6 months or ≥10% over >6 months.
2 Low Body Mass Index (BMI): ≤20 kg/m² (≥70 years) or ≤18.5 kg/m² (<70 years).
3 Reduced Muscle Mass (Skeletal Muscle Index [SMI]): calculated as appendicular skeletal muscle mass (measured via bioelectrical impedance analysis [25]) divided by height squared (kg/m²). The diagnostic cutoffs were ≤7.0 kg/m² for men and ≤5.8 kg/m² for women [26].

#### 2.3.2 Tongue pressure

Tongue pressure was measured (TPM-01; JMS, Japan) by placing a balloon between the tongue dorsum and palate. Participants were instructed to press maximally for approximately 7 s. After three trials (with approximately 30-s intervals), the highest value (kPa) was recorded [27,28].

#### 2.3.3 Other Variables/Covariates

Age, sex, Barthel Index [29], Mini-Mental State Examination (MMSE) [30], Charlson Comorbidity Index (CCI) [31], FOIS [23], and occlusal support status (Eichner classification [32,33]) were extracted from electronic records. Age, sex, MMSE, occlusal support status, and CCI were included as covariates in the CLPM/logistic models.

### 2.4 Oral rehabilitation and nutritional counseling

All participants in this study received oral rehabilitation and nutritional counseling. Generally, participants attended the clinic once every 2 weeks. However, actual intervals between visits were flexibly adjusted based on the severity of dysphagia and individual social circumstances (e.g., difficulty travelling to the clinic or availability of caregiving support).

• Oral Rehabilitation

Based on each participant’s oral health status and swallowing function, individualized rehabilitation plans were developed. Subsequently, dentists and dental hygienists specializing in oral rehabilitation provided approximately 30-min sessions, including direct training (exercises using food), indirect training (e.g., tongue exercises), and instruction on compensatory swallowing techniques.

• Nutritional Counseling

A registered dietitian developed a nutritional care plan based on a comprehensive assessment of dietary intake, body weight, and muscle mass. High-energy, high-protein dietary strategies were tailored to each participant’s swallowing ability. Nutritional counseling was provided during outpatient visits to monitor nutritional status.

### 2.5 Statistical analysis

All statistical analyses were performed using R (version 4.4.2), with significance set at 5% (two-tailed). The Wilcoxon rank-sum test (for continuous variables) and Fisher’s exact test (for categorical variables) compared baseline data between completers and dropouts. Descriptive statistics are reported as mean ± standard deviation or frequencies (%). Sample size adequacy was based on the requirement of at least five cases per CLPM parameter.

Changes over time in tongue pressure, SMI, and BMI were analyzed using one-way repeated-measures ANOVA. Greenhouse-Geisser correction was applied if sphericity assumptions were violated. Post-hoc paired t-tests with Holm-Bonferroni adjustment were applied where significant effects were found. McNemar’s test assessed changes in malnutrition prevalence.

A CLPM served as the primary analysis to examine bidirectional associations between tongue pressure and malnutrition, including autoregressive and cross-lagged paths, plus contemporaneous correlations. Age, sex, MMSE, occlusal support, and CCI were included as covariates. Standardized coefficients (β) are reported. Model fit was evaluated using the comparative fit index (CFI), Tucker–Lewis index (TLI), root mean square error of approximation (RMSEA), and standardized root mean square residual (SRMR), with thresholds of CFI ≥ 0.95, TLI ≥ 0.95, RMSEA ≤ 0.08, and SRMR ≤ 0.08 [34].

Logistic regression tested whether baseline tongue pressure predicted malnutrition at 6 and 12 months using the same covariates. Model fit was assessed by the Akaike Information Criterion (AIC). Results are presented as odds ratios (OR) with 95% confidence intervals (CI).

As a supplementary analysis, participants were stratified into two groups based on whether their tongue pressure had improved by ≥3 kPa or <3 kPa at 12 months, referencing thresholds used in previous studies.

Malnutrition prevalence was then compared at 12 months between the two groups using Fisher’s exact test.

## 3 Results

### 3.1 Participant characteristics

Of the 203 participants enrolled, 177 (87.2%) completed the 12-month follow-up. Attrition was due to death (n = 3), hospitalization (n = 2), and loss to follow-up (n = 21). No significant differences in baseline characteristics were observed between completers and dropouts (Table 1).

**Table 1:** Participant characteristics.

Characteristics	Completed N = 177^1^	Dropped out N = 26^1^	p-value^2^
Age, years	78.9 ± 7.3	80.6 ± 6.9	0.255
Sex, Female	70 (39.5%)	12 (46.2%)	0.527
Charlson Comorbidity Index			0.586
Low (0)	35 (19.8%)	7 (26.9%)	
Medium (1–2)	120 (67.8%)	17 (65.4%)	
High (≥3)	22 (12.4%)	2 (7.7%)	
Eichner classification^3^			0.166
Group A	71 (40.1%)	10 (38.5%)	
Group B	63 (35.6%)	5 (19.2%)	
Group C	43 (24.3%)	11 (42.3%)	
Cognitive Score	25.8 ± 4.2	26.8 ± 3.2	0.360
Barthel Index	82.1 ± 23.6	76.7 ± 28.1	0.587
RSST^4^ (<3)	113 (63.8%)	18 (69.2%)	0.665
FOIS^5^			0.967
1	6 (3.4%)	1 (3.8%)	
2	5 (2.8%)	1 (3.8%)	
3	2 (1.1%)	0 (0.0%)	
4	46 (26.0%)	7 (26.9%)	
5	42 (23.7%)	5 (19.2%)	
6	76 (42.9%)	12 (46.2%)	
7	0 (0%)	0 (0%)	
Tongue Pressure, kPa	21.1 ± 9.1	20.5 ± 8.6	0.827
Unintentional Weight Loss	11(6.2%)	2 (7.7%)	0.674
BMI, kg/m²	19.8 ± 3.1	20.7 ± 2.7	0.108
SMI, Male, kg/m²	6.42 ± 0.87	6.35 ± 0.81	0.751
SMI, Female, kg/m^2^	5.27 ± 0.74	5.35 ± 0.70	0.732
Malnutrition	130 (73.4%)	17 (65.4%)	0.483

Abbreviations: RSST, Repetitive Saliva Swallowing Test; FOIS, Functional Oral Intake Scale; BMI, Body Mass Index; SMI, Skeletal Muscle Index.

1 Values are presented as mean ± standard deviation or numbers (%).

2 Continuous variables were analyzed using the Wilcoxon rank-sum test; categorical variables were analyzed using Fisher's exact test.

3 Eichner classification: Group A (complete occlusal support), Group B (reduced occlusal support), Group C (no occlusal support).

4 RSST <3 indicates suspicion of dysphagia.

5 FOIS: 1： No oral intake, 2： tube dependent with minimal oral intake, 3： tube supplements with consistent oral intake, 4： total oral intake of single consistency, 5： total oral intake of multiple consistencies requiring special preparation, 6： total oral intake with specific food limitations (no texture modification needed), 7: total oral intake without restrictions.

### 3.2 Changes over time

Table 2 shows changes in key indicators over time. Tongue pressure increased significantly from baseline to 6 and 12 months (p < 0.001), while SMI decreased at 12 months (p < 0.05). BMI decreased from baseline to 12 months and from 6 to 12 months (both p < 0.001). Malnutrition prevalence increased significantly at 12 months (p < 0.05).

**Table 2:** Longitudinal changes in key variables^1^.

Variable	Baseline	6 months	12 months	p-value
Tongue Pressure (kPa)	21.1 ± 9.1	22.5 ± 9.3*	23.0 ± 9.4*, †	<0.001 §
SMI (kg/m²)	5.98 ± 0.99	5.95 ± 1.01	5.92 ± 1.02*	0.015 §
BMI (kg/m²)	19.8 ± 3.1	19.8 ± 3.0	19.5 ± 3.0*, †	<0.001 §
Malnutrition, n (%)	130 (73.4%)	134 (75.7%)	142 (80.2%)*	0.004 §

Abbreviations: SMI, Skeletal Muscle Index; BMI, Body Mass Index.

1 Values are presented as mean ± standard deviation or numbers (%).

* Significantly different from baseline (p < 0.05, after Holm-Bonferroni correction).

† Significantly different from 6 months (p < 0.05, after Holm-Bonferroni correction).

§ Statistically significant (p < 0.05); p-values for continuous variables from repeated measures ANOVA with Greenhouse-Geisser correction; for malnutrition from McNemar's test.

### 3.3 CLPM

**Fig. 2:**
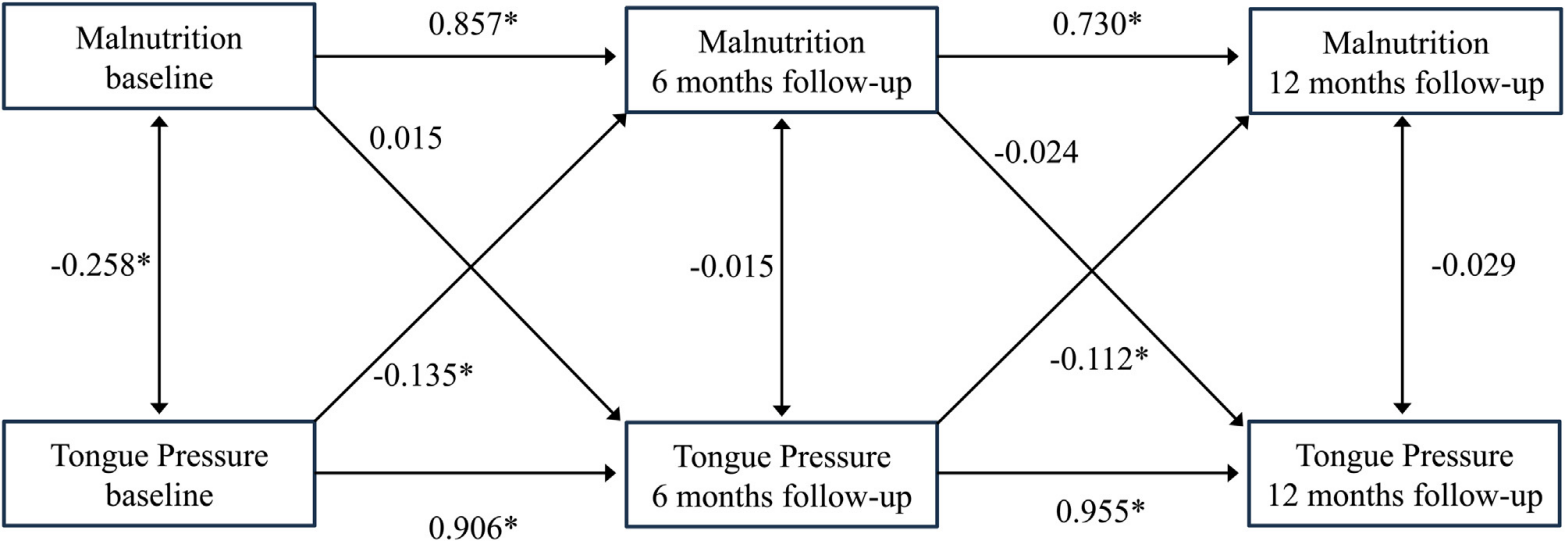
Cross-lagged panel model. Values represent standardized coefficients (β). *Indicates statistical significance (p < 0.05). Models are adjusted for age, sex, cognitive function, occlusal support classification, and comorbidity index. Model fit index: RMSEA = 0.067; SRMR = 0.051; CFI = 0.980; TLI = 0.964.

The CLPM showed good fit (RMSEA = 0.067; SRMR = 0.051; CFI = 0.980; TLI = 0.964) (Fig. 2).

Autoregressive coefficients indicated high temporal stability for both tongue pressure (baseline to 6 months: β = 0.906; 6 to 12 months: β = 0.955) and malnutrition (baseline to 6 months: β = 0.857; 6 to 12 months: β = 0.730) (all p < 0.001).

Tongue pressure showed a significant cross-lagged association with malnutrition (baseline to 6 months: β = −0.135, p < 0.001; 6 to 12 months: β = −0.112, p = 0.028), while malnutrition showed no significant cross-lagged association with tongue pressure　(baseline to 6 months: β = 0.015, p = 0.656; 6 to 12 months: β = −0.024, p = 0.280). Among covariates, malnutrition at 6 months was significantly affected by only female sex (β = 0.088, p < 0.01).

### 3.4 Logistic regression for malnutrition

Table 3 presents the results of the logistic regression analysis. Tongue pressure was significantly associated with malnutrition in both models (6 months: OR = 0.91, 95% CI: 0.86–0.95, p < 0.001; 12 months: OR = 0.89, 95% CI: 0.84–0.94, p < 0.001). Female sex was associated with higher risk at 6 months (OR = 2.47, 95% CI: 1.10–5.87, p = 0.033), while other covariates were not significant. The AIC values were 175.25 (6 months) and 157.09 (12 months).

**Table 3:** Logistic regression analysis for malnutrition at 6 and 12 months.

	6-month malnutrition		12-month malnutrition	
Characteristic	OR (95% CI)^1^	p-value	OR (95% CI)^1^	p-value
Tongue Pressure (kPa)	0.91 (0.86–0.95)	<0.001	0.89 (0.84–0.94)	<0.001
Age (years)	1.01 (0.95–1.07)	0.730	0.99(0.93–1.05)	0.731
Sex (Female)†	2.47 (1.10–5.87)	0.033	1.85(0.78–4.62)	0.172
Cognitive Score	0.97 (0.86–1.07)	0.713	1.02(0.91–1.13)	0.753
Eichner classification				
Group A	Reference		—	
Group B	0.74 (0.30–1.86)	0.528	0.87(0.32–2.30)	0.773
Group C	0.67 (0.23–1.97)	0.459	0.83(0.26–2.71)	0.748
Charlson Comorbidity Index	1.33 (0.93–2.00)	0.114	1.40(0.94–2.20)	0.121

1 OR = Odds Ratio, CI = Confidence Interval.

† Reference: Male.

### 3.5 Subgroup analysis

In the supplementary analysis, participants were stratified into two groups according to whether their tongue pressure had improved by ≥3 kPa or <3 kPa at 12 months, based on prior research.

Malnutrition prevalence at 12 months was compared between the two groups using Fisher’s exact test, and no statistically significant difference was observed (p = 0.428).

## 4 Discussion

In this study, 177 older adults with dysphagia were followed up for 12 months to examine the temporal associations between reduced tongue pressure and malnutrition. The CLPM demonstrated a significant cross-lagged association from tongue pressure to malnutrition at both 6 and 12 months, but not vice versa. Despite improvements in tongue pressure over time, malnutrition prevalence increased. Logistic regression analysis also indicated that lower baseline tongue pressure was significantly associated with malnutrition at both 6 and 12 months. These findings suggest that reduced tongue pressure may be an early indicator of malnutrition risk in older adults with dysphagia rather than a direct outcome of malnutrition. Furthermore, female sex was significantly associated with malnutrition at 6 months, aligning with previous studies that reported higher susceptibility among older women [35].

Previous studies have also identified pathways by which reduced tongue pressure may reduce food intake and energy consumption, potentially exacerbating malnutrition [11,12]. Theoretically, a reverse relationship, wherein malnutrition reduces tongue pressure through a reduction in overall muscle mass [13,14], could exist. However, no evidence of this relationship was found in our study. In older adults with severe dysphagia, reduced tongue pressure may limit food intake relatively early, whereas detecting the impact of malnutrition on tongue pressure may require an extended observation period. Furthermore, because the tongue musculature includes a high proportion of type I fibers [36], which may recover more readily with training [37], oral rehabilitation may contribute to the observed improvements in tongue pressure.

Multiple factors may contribute to the progression of malnutrition despite improvement in tongue pressure. First, 73% of the participants were already undernourished at baseline. For older adults, advanced malnutrition becomes irreversible [7]. Second, the improvement in tongue pressure observed in this study (approximately 2 kPa) did not reach the clinically significant threshold for improving swallowing function (3–5 kPa) [28,38]. Although dysphagia-modified diets are intended to ensure safety, they often lead to energy and protein dilution owing to the addition of liquids [39]. This, combined with potential issues such as poor compliance, may hinder adequate nutritional intake.

Further, our stratified analysis supported that even participants who achieved clinically significant improvements in tongue pressure (≥3 kPa) showed no significant difference in malnutrition prevalence at 12 months compared to those with minimal or no improvement. This finding suggests that improvements in tongue pressure alone do not automatically lead to better nutritional outcomes in older adults with dysphagia, pointing to the complex, multifactorial nature of malnutrition in this population and the need for integrated intervention strategies.

The temporal relationship between tongue pressure and malnutrition observed in this study may reflect part of the frailty cycle proposed by Fried et al. [40]. Specifically, the pathway from decreased tongue pressure to malnutrition suggests an aspect of the multifaceted interactions between physical function decline and sarcopenia characteristic of physical frailty. While these findings indicate that modest improvements in tongue pressure alone may not immediately translate to improved nutritional status, oral function training remains important not only for addressing malnutrition but also for preventing aspiration pneumonia [41]. Therefore, rehabilitation professionals should consider multidisciplinary collaboration with nutritionists besides oral function training and oral health management. In the future, developing integrated management systems that combine oral function training with nutritional guidance would be beneficial.

Furthermore, dysphagia associated with neurodegenerative diseases tends to progress irreversibly [42], potentially limiting the effectiveness of training interventions. In these conditions, flexible approaches tailored to disease characteristics—such as alternative swallowing techniques or food texture modifications—are required [43]. Depending on the underlying disease, early multidisciplinary collaboration and proactive nutritional management should be considered.

This study has some limitations. First, owing to its retrospective design, intervention details and measurement timing could not be standardized. Although the CLPM was employed, the potential for inferring causal relationships remains limited. Moreover, all participants underwent oral rehabilitation and nutritional counseling, but the specific interventions, adherence, and visit frequency were not uniformly tracked. This lack of consistent data collection may have masked or attenuated the association between tongue pressure and malnutrition in some cases. Second, the study population consisted exclusively of older adults with dysphagia, and most of them had a high baseline risk of malnutrition (73% at enrollment). Although the severity of dysphagia varied (FOIS scores ranging from 1 to 6), this was still a specialized and relatively homogeneous group compared to the broader geriatric population. Consequently, this study’s findings may not be fully generalizable to older adults without dysphagia. Moreover, excluding cases lacking complete assessment data at all three time points may have introduced selection bias. Third, we did not assess other oral functions beyond tongue pressure (e.g., chewing ability), and multiple assessors raised a potential risk of inter-rater bias. Fourth, malnutrition was defined based solely on phenotypic criteria without incorporating the etiological criteria from the GLIM framework. This may have led to an underestimation or overestimation of malnutrition prevalence. Lastly, we did not control for confounding factors such as medications, specific disease etiologies, socioeconomic status, or possible sex-specific differences due to sample size constraints and concern over model complexity. Future prospective research should capture detailed nutrition interventions, adherence levels, and oral function measures to clarify these relationships more accurately.

This study’s findings suggest that tongue pressure measurement could serve as a valuable tool for screening and longitudinal monitoring of older adults with dysphagia. We propose a staged intervention model based on tongue pressure values: for mild reductions, oral function training with regular observation; for moderate reductions, collaboration with nutritionists; and for severe reductions, intensive multidisciplinary intervention. Regular tongue pressure measurement may serve a dual purpose—enhancing patients' motivation for rehabilitation while functioning as an early alert system for healthcare providers. In clinical settings, we recommend regular tongue pressure assessment for older adults with dysphagia, combined with comprehensive care through stage-appropriate multidisciplinary collaboration.

## 5 Conclusions

This study, based on longitudinal analysis using the Cross-Lagged Panel Model, showed that decreased tongue pressure may precede malnutrition in older adults with dysphagia. However, given the retrospective observational design, causal relationships should be interpreted cautiously. To prevent nutritional decline, a comprehensive approach integrating oral rehabilitation and nutritional management is recommended.

## CRediT authorship contribution statement

HF: Conceptualization, data collection, analysis, investigation, methodology, visualization, writing (original draft).

YY: Methodology, project administration, validation, writing (proofreading and editing).

HF, YY, MO: Data collection, investigation.

TK, FT: Conceptualization, project administration, supervision, writing, proofreading and editing.

## Ethics approval and consent to participate

The Institutional Review Board and Hospital Research Ethics Committee approved the study protocol (No.NDU-T2017-41; approved date 1 July 2017). Owing to the retrospective design of the study, the requirement for patient consent was waived.

## Funding

This research did not receive any specific grant from funding agencies in the public, commercial, or not-for-profit sectors.

## Declaration of competing interest

The authors declare no conflict of interest.
